# Immunobiology of the biliary tract system

**DOI:** 10.1016/j.jhep.2022.08.018

**Published:** 2022-09-16

**Authors:** Niklas K. Björkström

**Affiliations:** Center for Infectious Medicine, Department of Medicine Huddinge, Karolinska Institutet, Karolinska University Hospital Huddinge, Stockholm, Sweden

**Keywords:** structural immunology, cholangiocytes, macrophages, innate lymphocytes, unconventional T cells

## Abstract

The biliary tract is a complex tubular organ system spanning from the liver to the duodenum. It is the site of numerous acute and chronic disorders, many of unknown origin, that are often associated with cancer development and for which there are limited treatment options. Cholangiocytes with proinflammatory capacities line the lumen and specialised types of immune cells reside in close proximity. Recent technological breakthroughs now permit spatiotemporal assessments of immune cells within distinct niches and have increased our understanding of immune cell tissue residency. In this review, a comprehensive overview of emerging knowledge on the immunobiology of the biliary tract system is provided, with a particular emphasis on the role of distinct immune cells in biliary disorders.

## Introduction

The biliary tract system consists of intra- and extrahepatic bile ducts and the gallbladder. It is a tubular organ system connected to the intestine via the duodenum that also closely interacts with both the liver parenchyma and the vascular system. Cholangiocytes (biliary epithelial cells) line the bile duct lumen.^1^ These cells are transcriptionally diverse, depending on their anatomical localisation (intra- vs. extrahepatic vs. gallbladder).^2^ Many immune cells are also present in close conjunction with the bile duct, making up an intricate cooperative machinery that defends against pathogens in healthy individuals. However, this machinery can also have a pathogenic role during acute and chronic bile duct diseases.

Cholangiopathies/chronic cholestatic liver diseases can be divided into genetic, such as Alagille syndrome and cystic fibrosis-associated liver disease, and idiopathic/multifactorial diseases, including biliary atresia, primary biliary cholangitis (PBC), and primary sclerosing cholangitis (PSC).^1,3–5^ While many of these diseases likely share downstream pathogenic traits, initial insults for idiopathic diseases remain largely elusive. What is clear is that cholangiocytes are the primary shared targets in these diseases and that the immune system plays a central role in their pathogenesis. Furthermore, if left untreated, patients with cholangiopathies risk developing end-stage liver disease, making liver transplantation the only viable option. Another threat for these patients is the development of cholangiocarcinoma, the second most common primary liver cancer, which is associated with a dismal prognosis.^6^ Although less is known regarding immune surveillance of cholangiocarcinoma, recent data suggest an important contribution of the tumour immune microenvironment to disease outcome.^7–9^

Microscopically, the liver is a highly stratified organ with liver lobules constituting the functional units. Cholangiocytes (and hence bile ducts) make up only 5% of all cells in the liver and are localised at defined regions within the liver lobule (portal tracts). Liver immune cell composition varies throughout the liver lobule. This implies that taking tissue architecture into account will be paramount for understanding the immunobiology of the liver and biliary tract system in health and disease. Herein, recent insights into the spatial organisation of the liver and biliary immune landscape will be discussed with a focus on human immunology when possible. Based on this knowledge, the role of biliary and/or intrahepatic immune cells in cholangiopathies will be addressed. Finally, unresolved issues will be discussed, and open questions to be answered in future studies outlined.

## Structure of the liver and biliary tract immune system

### Technological development enables single-cell spatial resolution

Cells need to be correctly spatially arranged for an organ to function. Low-dimensional microscopy techniques (immunohistochemistry, immunofluorescence), conventional flow cytometry, and bulk array- or sequencing-based methods for transcriptomic assessment have been available for decades, partially enabling such spatial localisation in immunological research of bile duct diseases. However, the capacity to gain in-depth biological data on immune cells in tissues from limited clinical material, such as liver biopsies and/or liver fine-needle aspirates, laser micro-dissected tissue areas, or other biological material (including brush samples taken during endoscopic retrograde cholangiopancreatography [ERCP] procedures), has been hampered by technical limitations. Furthermore, since the liver lobule is structurally highly organised and intrahepatic bile ducts occupy a distinct spatial niche, the absence of technologies allowing for high-dimensional data acquisition in a spatial context has remained an obstacle. Thus, the development seen over the last couple of years has truly revolutionised our capability to map human immune and non-immune cells in tissues at the single-cell level. Flow cytometry has moved from simultaneous assessment of a few markers to >20 parameter instruments, and specialised units are pushing beyond 30 parameters with CyTOF (cytometry by time of flight) offering a second viable alternative.^10,11^ Furthermore, the release of Smart-Seq2 had a significant impact on our capacity to perform single-cell RNA sequencing (scRNAseq),^12^ and more recent droplet-based approaches have made it possible to analyse a high number of cells in parallel.^13^ Usage of scRNAseq, together with mapping and microscopy approaches, recently provided unprecedented insights into liver lobule zonation, suggesting the existence of multiple zones with distinct cell composition.^14,15^ Public scRNAseq data now exists for all immune cells and specific immune cell subpopulations from bile duct disorders, such as PSC^16^ and biliary atresia,^17^ as well as cholangiocarcinoma.^18^ The advent of spatial transcriptomics,^19,20^ although not yet technically providing data at the single-cell level, has taken this even further.^21,22^ In parallel, novel microscopy techniques now allow for simultaneous assessment of >50 parameters.^21,23^ Applying this paradigm-shifting development to biliary diseases, combined with a thorough sampling of biological specimens, holds significant potential for the future (the application of these technologies in liver research was recently reviewed in^24^). However, increased data granularity, for instance from scRNAseq of intrahepatic myeloid cells, leading to identification of novel subpopulations, might also make it necessary to revisit and challenge existing conventions. There will also be a need for consolidation in the field concerning definitions of old and new subtypes of immune cells. The upcoming sections will discuss immune cell tissue residency and the spatial organisation of immune cells in the liver parenchyma and, more specifically, in relation to the biliary tract system.

**Key point**

Recent technological development now allows for single-cell (spatial) resolution of immune cells in tissues.

**Key point**

The liver is enriched with innate immune cells including specialised macrophages, NK cells, and unconventional T cells, many of which appear to be tissue resident.

### Immune cell tissue residency

To comprehend the immunobiology of the biliary tract system, we first need to understand immune cell access to tissues and their recirculation patterns. While it has been known for a long time that specialised types of macrophages exist in different peripheral organs, *e.g*. Kupffer cells in the liver, a common notion until a decade ago was that most lymphocytes would continuously move from the circulation into peripheral organs and recirculate via lymphatics. However, it is now clear that distinct lineages of tissue-resident lymphocytes also exist, such as tissue-resident memory T cells, innate lymphoid cells (ILCs), and tissue-resident natural killer (NK) cells.^25,26^ We have also learnt from parabiosis studies in mice that surface proteins, such as CD69, CD49a, and CD103 can be used to identify tissue-resident cells.^27,28^ These proteins exhibit functional roles in retaining immune cells in tissues (reviewed in^25,26^). The validity of such tissue-residency markers has also been confirmed in humans in clinical organ transplantation settings (involving the liver, gut, lung, and uterus), albeit with a higher rate of replenishment for certain immune cell types in human organs, possibly because of a different inflammatory tone and/or environmental exposure at steady state compared to mice.^29–34^ With the realisation that large fractions of immune cells permanently reside in peripheral organs, we have started to understand their frontline role in defence against microbes, inflammatory processes, and in combatting tumours.^35–39^ These tissue-resident cells also orchestrate the recruitment of circulating immune cells to tissues.^35–38^ Given the complex architecture of the liver and biliary tract system, knowledge of immune cell tissue-residency patterns will be critical to consider. Recent technological developments have also provided us with new means to study the spatial organisation of immune cells.

### The liver immune landscape

**Fig. 1:**
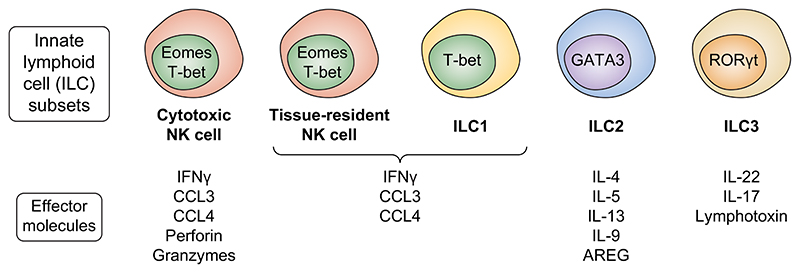
Overview of NK cell and ILC subsets including master transcription factors regulating these cells and the key effector cytokines they produce. Unlike T and B cells, ILCs do not express highly variable antigen receptors. Below, the major 1LC subsets are introduced (previously reviewed in detail here:^26,109^). NK cells are cytotoxic and proinflammatory (release IFNγ, TNF, and chemokines such as CCL3, CCL4, and CCL5) ILCs that are prevalent in the circulation and enriched in certain peripheral tissues such as the liver. They are defined as CD56^+^CD3^-^ lymphocytes and express the master transcription factors Eomes and T-bet. ILC1s are cytokine-producing cells (IFNγ) defined by expressing the master transcription factor T-bet while lacking Eomes. A mouse-human species difference for ILC1s is that they are prevalent in mouse liver tissue (NK1.1^+^CD49a^+^CD49b^−^ cells) whilst the human functional counterpart would be CD56^bright^CD16^−^ liver-resident NK cells. 1LC2s express the master transcription factor GATA3 and exhibit Th2 cytokine responses. 1LC3s are identified by the master transcription factor RORγt and have the capacity to produce both IL-17 and IL-22. AREG, amphiregulin; CCL, C–C motif chemokine ligand; Eomes, eomesodermin; GATA3, GATA binding protein 3; 1FN, interferon; 1L, interleukin; 1LCs, innate lymphoid cells; NK, natural killer; RORγt, retinoid orphan receptor-γt.

**Fig. 2:**
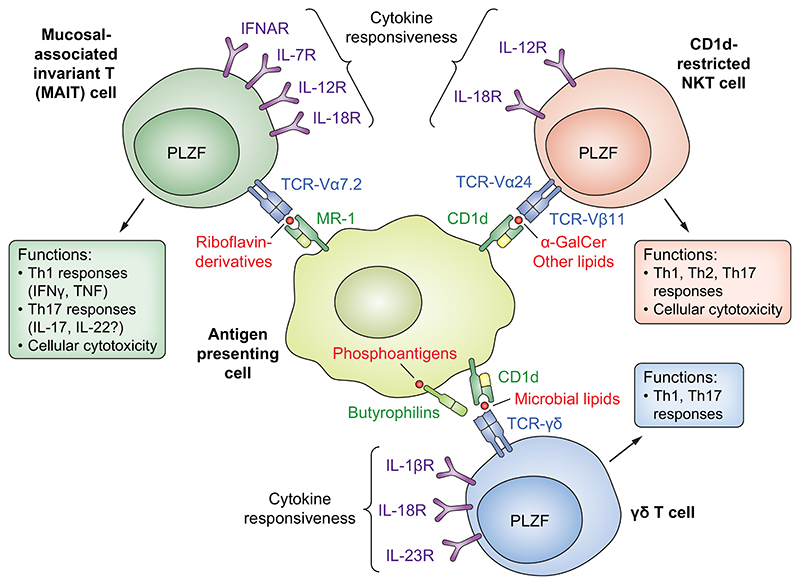
Overview of major unconventional T-cell populations, their cytokine responsiveness, TCR restriction, ligands, and major effector functions. Below, the central unconventional T-cell populations are introduced (previously reviewed in detail here:^110^ MAIT cells are defined by the expression of a 5-OP-RU tetramer or co-expression of TCR-Va7.2 and CD161 and recognise vitamin B2 (riboflavin) metabolites presented on the non-polymorphic MR1. MAIT cells are highly enriched in the human liver but scarce in mouse liver. They exhibit Th1 (IFNγ) and Th17 (IL-17) immunity in response to bacterial infections or proin-flammatory cytokines. γδ T cells represent a distinct T-cell linage expressing a TCR that can recognise a wide array of exogenous and endogenous molecules such as bacterial toxins, microbial lipids (via CD1d), viral proteins, and phosphoantigens (via butryophilins). γδ Tcells exhibit proinflammatory Th1 and Th17 functions that can either be protective or pathogenic during immune responses. γδ T cells are enriched in the human liver. CD1d-restricted NKT cells display an invariant TCR (typically Va24 paired with Vb11) that recognises glycolipids presented on CD1d. These cells have been called a “Swiss-army knife” of the immune system having the capacity to produce a broad range of Th1, Th2, and Th17 cell-associated cytokines. CD1d-restricted NKT cells are highly prevalent in mouse liver but scarce in human liver. IFN, interferon; IL, interleukin; MAIT, mucosal-associated invariant T; MR1, MHC class I related-1 molecule; NKT, natural killer T; PLZF, promyelocytic leukaemia zinc finger (or ZBTB16); TCR, T-cell receptor; Th, T helper; TNF, tumour necrosis factor.

Although most immune cell subsets can be found in the liver, innate immune cells are specifically enriched in this organ compared with the circulation.^26,39–44^ One of the most prevalent lymphocyte populations in the liver is a subset of ILCs called NK cells. These liver-resident NK cells are characterised as CD56^bright^CD16^-^ NK cells in humans,^45,46^ or CD49a^+^CD49b^-^NK1.1^+^ ILC1s in mice,^47^ and have diminished cytotoxic potential compared to conventional CD56^dim^CD16^+^ NK cells but efficiently respond with proinflammatory cytokines and chemokines such as interferon (IFN)γ, tumour necrosis factor, C–C motif chemokine ligand (CCL)3, CCL4, and CCL5 upon activation and/or target cell recognition.^26,48^ Other non-NK-ILCs are also present in the human liver, but their numbers and exact location have been less well studied (Fig. 1).^41^ Also, certain types of unconventional T cells are prevalent in liver tissue (Fig. 2). In humans, these include mucosal-associated invariant T (MAIT) cells and γδ T cells,^44,49^ and in mice CD1d-restricted NKT cells.^50^

Liver-resident NK cells, MAIT cells, and γδ T cells share high expression of the tissue-residency marker CD69 and the liver-homing C-X-C motif chemokine receptor (CXCR6),^44–46^ a subset of liver-resident NK cells express CD49a,^45^ but all these cells display low CD103 expression. γδ T cells appear to be evenly distributed throughout the liver lobule, while MAIT cells are enriched in portal tracts.^44,51^

Conventional TCR_αβ_^+^ T cells within the liver are enriched for memory T cells compared with peripheral blood, and the liver CD4/CD8 ratio is skewed towards CD8 T cells.^52,53^ Most intrahepatic memory T cells express the tissue-residency marker CD69, while a smaller fraction also co-express CD103.^52,53^ Conventional CD4 and CD8 T cells are present throughout the liver parenchyma but appear enriched in portal areas, although more studies are needed to determine the exact localisation of T cells and their subsets within the liver lobule.^21^

**Fig. 3:**
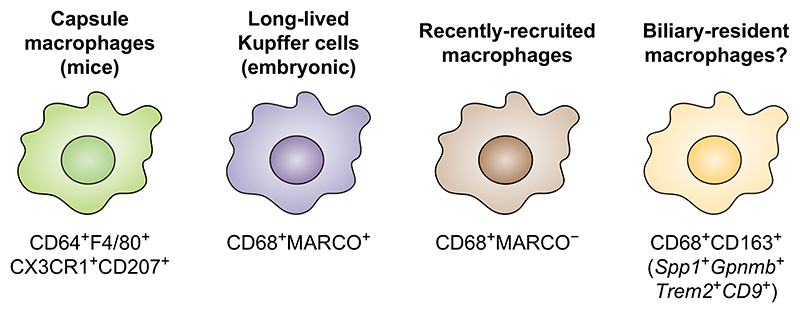
Overview of liver macrophages including major identifying surface markers. Historically, all macrophages residing in the liver were considered Kupffer cells. However, recent lineage tracing studies in mice and scRNAseq experiments in humans and mice, both in steady state and disease settings, have revealed considerable heterogeneity within the liver macrophage compartment (previously reviewed here:^39^). At steady state, human liver macrophages can roughly be divided into CD68^+^MARCO^+^ and CD68^+^MARCO^-^ subsets.^39,54,111^ Different subpopulations within these two main subsets have also been identified, and the composition changes in disease settings, including the appearance of scar-associated TREM2^+^CD9^+^ macrophages originating from monocytes.^111^ The CD68^+^MARCO^+^ subset corresponds to murine liver-resident long-lived Kupffer cells and is immunoregulatory while CD68^+^MARCO^-^ macrophages are recently recruited from blood and are more proinflammatory.^39,54,112^ Beyond this, in mice, capsule macrophages are also present55 but have not been reported in humans. Finally, the murine lipid-associated macrophages (Spp1^+^Gpnmb^+^Trem2^+^CD9^+^), recruited during metabolic inflammation, might represent a murine counterpart of scar-associated macrophages.^39,113^ CX3CR1, C-X3-C motif chemokine receptor 1; Gpnmb, glycoprotein nmb; MARCO, macrophage receptor with collagenous structure; scRNAseq, single-cell RNA-sequencing; Spp1, secreted phosphoprotein 1; Trem2, triggering receptor expressed on myeloid cells 2.

Several types of macrophages exist in human and murine livers (Fig. 3). Whereas long-lived CD68^+^MARCO^+^ Kupffer cells (and their murine counterpart) predominantly reside in periportal and mid-lobular areas in sinusoids, recently recruited CD68^+^MARCO^-^ macrophages are found in higher abundance in portal tracts around blood vessels.^21,54^ Furthermore, as the name implies, murine capsule macrophages are localised close to the liver capsule, where they sense and defend against peritoneal microorganisms.^55^

**Fig. 4:**
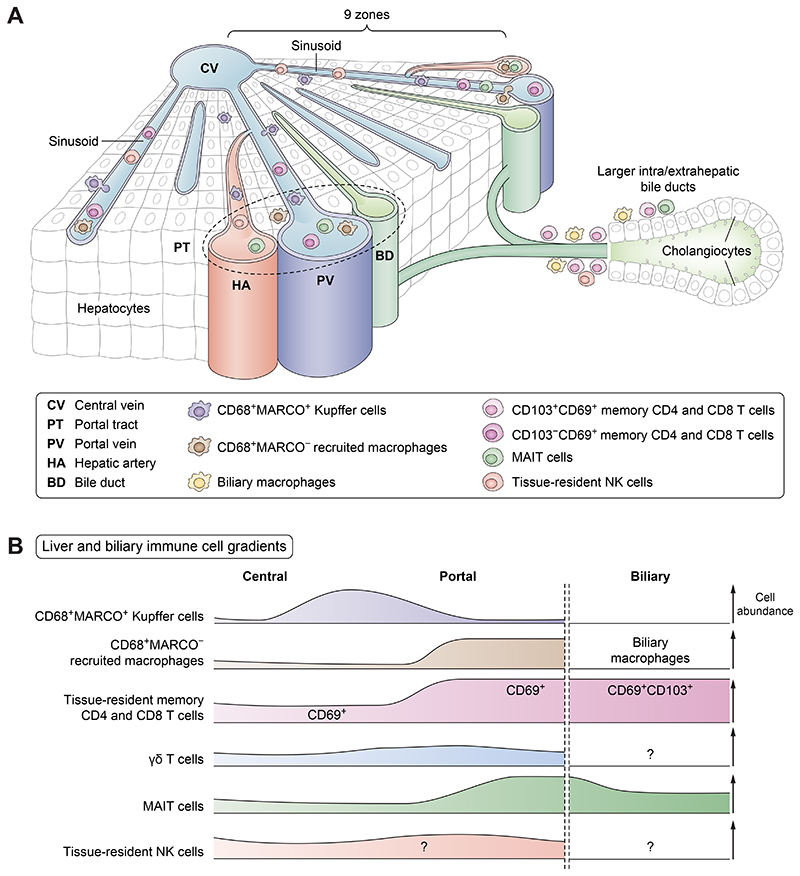
Structural immune cell gradients in the liver and bile ducts. (A) Schematic overview of a liver module and a bile duct including the spatial localisation of indicated myeloid and lymphoid cell types. (B) Gradients of immune cell presence at steady state in the liver lobule and in close proximity to intra- and extrahepatic bile ducts.

In summary, the immune composition in the liver is distinct from that in the circulation and an essential factor to consider in studies of liver and bile duct diseases. Although some unknowns remain, it is also evident that the immune landscape of the portal, periportal, and mid and central areas of the liver lobule is distinct in composition (Fig. 4).

**Key point**

The biliary immune niche is distinct in composition compared to the liver parenchyma and enriched for intra-epithelial resident memory CD8 T cells.

### Unique spatial immunological niche of the biliary tract system

Given the spatial restriction of larger intrahepatic bile ducts to portal tracts, it will likely be important to find means to assess the locally restricted immune compartment surrounding bile ducts at the microscopic level. Whereas we are starting to appreciate immune compartmentalisation within the liver lobule, less is known about the biliary tract system niche (Fig. 4). Nevertheless, recent work using “spatial sampling” (brush samples taken during ERCP procedures) and methods allowing for high-dimensional and spatial resolution has started to shed light on the biliary immune niche.^21,56^ While a significant fraction of liver lymphocytes express the tissue-residency marker CD69, fewer cells co-express CD69 and CD103.^52,56^ Instead, CD69^+^CD103^+^ lymphocytes are highly enriched around bile ducts.^56^ Although hepatocytes express low to intermediate levels of the CD103-ligand E-cadherin, cholangiocytes, lining both intra- and extrahepatic bile ducts, express high levels of E-cadherin.^56^ Thus, this molecule might be responsible for spatially retaining CD103-expressing lymphocytes close to bile ducts. A similar enrichment of CD69^+^CD103^+^ cells close to epithelial cells is seen in other human organs such as the intestine and lung.^57^ Cholangiocytes also produce transforming growth factor-β (TGFβ),^58^ which, together with interleukin (IL)-15, can promote the development and/or retention of CD69^+^CD103^+^ cells close to bile ducts.^59^

Regarding innate lymphocyte and unconventional T cells (Figs. 1 and 2), NK cells, MAIT cells, and γδ T cells are enriched in liver tissue, but they appear not to be equally enriched close to bile ducts.^56,60^ Although, at least for MAIT cells, more cells are found surrounding bile ducts than in the circulation.^60^ While these cells have been identified and enumerated close to bile ducts, limited data are available on the exact functions of biliary innate lymphocytes and unconventional T cells. Instead, most of the CD69^+^CD103^+^ immune cells within the biliary niche are tissue-resident effector memory CD8 T cells.^56^ These cells display a distinct TCR-repertoire and transcriptional profile compared with circulating effector memory CD8 T cells and produce IFNγ, IL-17, and IL-22 upon stimulation.^56^ CD4 T cells with a similar profile also reside close to bile ducts, although at lower numbers.^53,56^ Common for both CD4 and CD8 T cells is high expression of CXCR6 and C–C motif chemokine receptor (CCR)6,^53,56^ possibly contributing to recruitment of these cells to the liver. However, since other T cells outside the biliary niche also express these chemokine receptors, they are most likely not exclusively guiding these cells to the biliary niche. α4β7 integrin expression might also contribute to recruitment of immune cells to the biliary niche.^56^ In studies using brush samples taken during ERCP procedures, the overall lymphocyte composition was similar in intra- and extrahepatic bile ducts.

Although fewer monocytes and macrophages localise to the biliary niche compared to how prevalent they are in the blood, cells expressing CD68 and CD163 can be found close to cholangiocytes.^56^ A study using high-dimensional imaging combined with spatial transcriptomics recently revealed these CD68^+^CD163^+^ cells to be lipid-associated macrophages (*Spp1^+^Gpnmb^+^Trem2^+^CD9^+^*) (Fig. 3).^21^ This population of macrophages, present in both humans and mice, are likely not long-lived tissue-resident cells, but recruited monocytes from the circulation (possibly through interactions with fibroblasts mediated by CCL2 and CD44).^21^

Taken together, studies in recent years have started to outline the composition of the biliary immune niche (Fig. 4). However, much work remains to be performed concerning most of these immune cell populations, both at steady state and in settings of biliary tract diseases. Nevertheless, in the upcoming sections, more specific roles of distinct immune cells in different biliary diseases will be discussed, taking the spatial context into account when possible.

**Key point**

The exact composition of the biliary immune niche in the context of health and in biliary disorders still needs to be determined.

## Cholangiocytes and immune cells in biliary tract diseases

In the following sections, the immunobiology of acute and chronic inflammatory biliary diseases will be covered. The tumour immune microenvironment of cholangiocarcinoma has recently been reviewed elsewhere^6,61^ and will not be extensively covered.

### Cholangiocytes as initial sensors of stress

Lining the biliary tract system, cholangiocytes are at the forefront and can be activated by infectious, toxic, inflammatory, and autoimmune insults (reviewed in detail here^1^:). This activation of cholangiocytes leads to proinflammatory cytokine production, crosstalk with immune cells in the vicinity, and cholangiocyte proliferation. Although the exact nature of initial insults remains elusive for cholangiopathies such as PBC and PSC, downstream pathophysiological processes likely share many features, including chronic cholangiocyte activation, recruitment of immune and mesenchymal cells, cholestasis, inflammation, and fibrosis development. This complex cascade is referred to as the ductular reaction.

**Key point**

Cholangiocytes are early responders to stress and likely participate in driving biliary disorders by propagating subsequent immune responses.

Activated cholangiocytes have been shown to secrete proinflammatory and pro-fibrogenic factors such as IL-6, CCL2, and TGFβ.^58,62^ A study where laser microdissections of ductular reactions of patients with end-stage PSC were compared to patients with end-stage HCV reported increased gene expression of chemokines known to attract neutrophils (C-X-C motif ligand [CXCL]1, CXCL6, CXCL5, and CXCL8) in those with PSC.^1,63^ Interestingly, the chemokine CCL28, which can promote homing of CCR10-expressing lymphocytes, was specifically increased in early PSC.63 Cholangiocytes also constitutively produce CXCL16, a chemokine that can recruit CXCR6-positive lymphocytes, and this expression appears to increase in biliary diseases such as PBC and PSC.^64^ Similarly, cholangiocyte activation led to increased expression and production of C-X3-C motif chemokine receptor 1.^65^ Beyond having the capacity to recruit immune cells via release and/or trans-presentation of chemokines upon activation, cholangiocytes constitutively express specific adhesion molecules, *e.g*. E-cadherin, and can upregulate others after activation, *e.g*. vascular cell adhesion molecule 1 (VCAM-1).^56,66^ Thus, the initial sensing of stress (infectious, toxic, inflammatory, and autoimmune) by cholangiocytes initiates a potent proinflammatory and chemotactic programme. The consequences of this concerning immune cell homing to bile ducts and subsequent activation, are discussed in the following sections.

Beyond contributing to local inflammation and recruitment of immune cells via released factors, cholangiocytes can also directly interact with immune cells via receptor-ligand interactions. As examples of antigen-presentation capabilities, cholangiocytes express CD1d and MHC class I related-1 molecule (MR1).^8,67^ They can, via these MHC-class I-like receptors, present to and activate both CD1d-restricted NKT cells (mouse and human) and MAIT cells (human).^8,67^ Another group of stress-induced ligands are MICA/MICB that, together with other ligands in mice and humans, can be recognised by the activation receptor NKG2D (also known as KLRK1) expressed by CD8 T cells and NK cells. Cholangiocytes have been reported to upregulate MICA in response to parasitic infection,^68^ and NKG2D ligands are likely also induced in the rotavirus-induced biliary atresia model, since blocking NKG2D ameliorates disease.^69^

Since cholangiocytes appear to be transcriptionally distinct depending on their localisation,^2^ future work should attempt to determine if this translates into different activation profiles and/or inflammatory responses. Additionally, more detailed studies on the crosstalk between cholangiocytes and immune and stromal cells within the biliary niche are warranted.

### Neutrophils

Despite neutrophils being the most abundant leukocyte in peripheral blood, we have only recently started to appreciate their functional heterogeneity and complex roles in the orchestration of inflammation and tissue repair.^70^ While few neutrophils are found in non-inflamed bile ducts, they infiltrate the biliary microenvironment in patients with PSC.^56^ Their recruitment might, in part, be propelled by biliary-resident T cells since biliary neutrophil and tissue-resident T-cell numbers positively correlated in a large cohort of patients with PSC and CD8 T cells in bile ducts displayed a transcriptome skewed towards recruitment of neutrophils.^56^ Interestingly, CXCL8, the main chemokine for neutrophil recruitment, was elevated in the bile of patients with PSC, and its levels have also been shown to associate with PSC disease progression.^71,72^ Neutrophils might, in turn, promote pathogenic T helper (Th)17 cell differentiation. Indeed, in inflammatory bowel disease, neutrophils are a significant source of IL-23, which can promote Th17 cell differentiation.^70^ Another mechanism for biliary neutrophil recruitment was recently suggested in mice. It included a loss of tuft cells in extrahepatic bile ducts, ensuing cholangiocyte activation, and a possible CXCL5-mediated neutrophil recruitment mechanism.^73^ Although microbial signals were necessary for the biliary neutrophil influx in mice,^73^ biliary neutrophil numbers in patients with PSC were independent of prior cholangitis episodes and bile microbial composition.^56^ Spatially, neutrophils are positioned closer to cholangiocytes in patients with PSC than in controls.^56^ Neutrophils have also been shown to interact with cholangiocytes via intercellular adhesion molecule 1 and VCAM-1, contributing to cholestasis in patients with alcoholic hepatitis.^74^ However, exactly how neutrophils contribute to biliary disorders remains to be determined.

**Key point**

Neutrophils and Th17 cell responses might cooperate in PSC to drive disease.

### Mononuclear phagocytes

Monocytes and macrophages have been extensively studied in cholangiopathies (recently reviewed here^42^:). A challenge in the field will be to incorporate a wealth of pathogenesis studies, both in mice and humans, into recently refined paradigms of liver macrophage heterogeneity (Fig. 3, discussed above). Beyond this, factors such as origin (foetal *vs*. bone marrow-derived), local environment (spatial confinement close to bile ducts), type of inflammation and/or model (acute vs. chronic), and time (early or late disease), will be key points to consider when evaluating macrophages in bile duct diseases.^38^ Nevertheless, as a general concept, monocytes and macrophages are responsive to both cholangiopathy-associated dysbiosis (microbes, microbial compounds) and bile acids.^75,76^ Such activated myeloid cells could promote cholangiocyte activation and proliferation.^75,76^ Liver macrophages express the bile acid-sensing receptor TGR5 (also known as GPBAR1).^42^ In this context, it is of interest that TGR5 is upregulated in CD68+CD206+ macrophages from liver tissue explanted from patients with PSC; this likely reflects a changing cytokine expression profile in these cells.^77^ Whether or not liver macrophages also respond functionally via the nuclear bile acid receptor FXR (farnesoid X receptor) remains to be determined. Future dual targeting of TGR5 and FXR in mouse models (macrophage-specific knockouts) or in *in vitro* systems might help to elucidate the possible contribution of these cells in driving the disease downstream of toxic bile.

In acute and chronic sclerosing cholangitis models, monocytes are recruited to the liver in a CCR2-dependent fashion, subsequently differentiate into macrophages with a proinflammatory phenotype, and finally localise to the peribiliary area.^78^ Genetic deletion of *Ccr2* attenuated the accumulation of monocytes and ameliorated overall disease progression.^78^ A similar mechanism was shown in another mouse model of acute cholangiocyte injury, with cholestasis, CCR2-dependent monocyte recruitment, and induction of avβ6 integrin expression on cholangiocytes, the latter driving cholangiocyte proliferation.^76^ Corroborating this, accumulation of myeloid cells has been noted in livers from individuals with end-stage PSC.^76,78^ On the other hand, another study relying on bile duct brush samples from patients with moderate to advanced, but not end-stage, PSC showed no apparent increase in myeloid cells in close proximity to cholangiocytes.^56^ Thus, the disease stage needs to be considered for future work evaluating the role of monocytes. Although more is known about macrophages in PSC compared to PBC, in a mouse model of PBC they appear to regulate NK cell responses via cytokines and possibly via crosstalk with the activating NK cell receptor NKG2D.^42^

In paediatric cholestatic liver diseases, a recent study performed scRNAseq on explanted liver tissue of patients with biliary atresia and Alagille syndrome.^79^ This revealed three populations of liver macrophages, largely overlapping with the main subsets of liver-resident long-lived CD68^+^MARCO^+^, recently recruited CD68^+^MARCO^-^ macrophages, as well as lipid-associated macrophages (Fig. 3).^79^ Although current understanding of Alagille pathogenesis, with defective NOTCH-signalling, revolves around cholangiocyte pathophysiology with incomplete development of intrahepatic bile ducts, populations of macrophages might play a role in downstream inflammatory events following cholestasis.^80^ Mononuclear phagocyte mapping in biliary atresia, using scRNAseq, identified 9 subsets of liver myeloid cells, including 4 monocyte/macrophage populations, and suggested a bile acid-driven hypo-inflammatory phenotype.^17^

Although much literature indicates roles for myeloid cells in acute and chronic cholangiopathies, future work would benefit from taking heterogeneity (resident *vs*. recruited), spatial localisation, and timing (acute *vs*. chronic, early disease *vs*. end-stage disease) into account when possible. Also, compared to monocytes and macrophages, few studies have assessed the role of dendritic cells in cholangiopathies.

### Adaptive lymphocytes

Much work has been performed on conventional CD4 and CD8 T cells, in both the circulation and liver tissue of patients with cholangiopathies, and in murine experimental models.^1,3,81^ Challenges in the field of human chronic progressive inflammatory diseases include accessing tissue early in the disease and explicitly studying local events close to bile ducts. Additionally, questions remain regarding the contributing role of tissue-resident T cells to disease compared with recently recruited cells. Nevertheless, with PSC as an example, a common finding has been elevated Th17 cell responses, both in blood and liver tissue.^75,82,83^ Neutrophils, monocytes, and cholangiocytes have been suggested to promote local Th17 cell differentiation/polarisation.^75,83,84^ A recent scRNAseq analysis of livers from patients with PSC also identified a naïve CD4 T-cell population close to intrahepatic bile ducts that displayed the potential to become Th17 cells.^16^ Similarly, biliary-resident CD8 T cells with the capacity to produce IL-17 are highly enriched close to the bile ducts in patients with PSC.^56^ IL-17 then promotes cholangiocyte activation and proliferation via JAK2-STAT3 signalling and subsequent disease progression.^84^ However, the pathogenic role of IL-17 was recently questioned in a murine model of cholangitis where IL-17 instead promoted PD-L1 expression on cholangiocytes and subsequently protected against CD8 T cell-mediated disease.^83^ Indeed, as an outlook, IL-17 receptor blockade appears to worsen intestinal disease activity in inflammatory bowel disease.^85^ Beyond IL-17 and Th17 cell responses in PSC, recent work in murine models of PBC and PSC suggested that the nature of Th cell responses modulates intrahepatic tumour immune surveillance, with Th1- and Th2-skewed responses favouring such surveillance.^86^

**Key point**

Recruited proinflammatory monocytes contribute to biliary disorders.

CD8 T cells with a mucosal phenotype accumulate around bile ducts in patients with PSC.^56^ Aberrant homing of such T cells from the gut to the bile duct is a prevailing hypothesis of PSC pathogenesis,^87,88^ where T cells express receptors such as CXCR6, CCR9, and a4b7 integrin^64,89^ and endothelia and/or epithelia upregulate MAdCAM-1 (mucosal vascular addressin cell adhesion molecule 1), CCL25, and E-Cadherin.^87^ However, recent work suggested this as a pan-aetiological phenotype in chronic liver diseases.^56,90^ This illustrates the necessity of conducting research on early disease pathogenesis, or in the case of PSC, possibly focusing on post-transplant recurrent PSC as a model for early pathogenesis studies. Interestingly, recent work on autoreactive pyruvate dehydrogenase complex E2-specific CD8 T cells in PBC demonstrated that these cells had an intraepithelial CD103^+^ tissue-resident memory phenotype and were localised close to intrahepatic bile ducts.^91^

**Key point**

Cholangiocytes can present antigens to several unconventional T-cell populations making these cells of interest for the study of biliary disorders.

A recent study on a family where 5 individuals suffered from PSC, identified a heterozygous missense mutation in *SEMA4D* (encoding Semaphorin-4D/CD100) in all 5 individuals, further linking T cells to PSC pathogenesis.^92^ This mutation was related to T-cell functional defects on the transcriptional and functional level in murine models and patient samples, and replacement by wild-type T cells in mice carrying the same disease-causing mutation as the sick family members attenuated cholangitis after DDC (3,5-diethoxycarbonyl-1,4-dihydrocollidine) exposure.^92^ Although the CD100-mutation was private to this family, it represents the first casual mutation leading to PSC. Future work should more broadly assess CD100 function and signalling and its associated pathways to evaluate the wider relevance of this finding in the entire PSC population.

Compared to T cells, our knowledge of the role of hepatic and biliary B cells in cholangiopathies remains scarce, even though antibodies and/or auto-antibodies likely play a role in IgG4-related hepatobiliary disease and PBC (reviewed in^93^), and despite the fact that IgA is the second most abundant protein in bile.^94^ Although it currently remains unclear whether the elevated IgG4 antibodies, by themselves, are pathogenic in IgG4-related cholangitis, elevated IgG4 levels can be found in other autoimmune, allergic, and infectious conditions and associate with type 2 immunity.^95^ The clinical association with blue-collar work suggests either molecular mimicry towards environmental factors and/or a more direct effect of these factors on inflammation.^96^ Interestingly, in a rotavirus model of biliary atresia, IgG autoantibodies were accumulated in the liver, and rituximab was efficient in eliminating hepatic B cells in patients, resulting in restored myeloid and T-cell function.^17^ In the same model, B cells were highly activated and produced cytokines promoting pathogenic T- and myeloid-cell responses.^97^ Recent work also compared B-cell receptor repertoires in the liver and gut tissue of patients with PSC.^98^ Nevertheless, future studies should focus on determining the exact types of B cells present within the liver and bile ducts of patients with cholangiopathies, their localisation, and the microenvironment. Methods such as IgA-SEQ or VirScan might be helpful to understand the reactivity of antibodies present in bile in relation to pathogenic microorganisms, normal flora, and dysbiosis.^99,100^

### Unconventional T cells and innate lymphocytes

As mentioned above, both NK cells and unconventional T cells, such as MAIT cells and γδ T cells, are enriched in healthy human liver tissue. At the same time, only MAIT cells are also enriched specifically around bile ducts.^56,60^ Both MAIT cells and γδ T cells are decreased in the liver parenchyma during chronic liver diseases, including biliary diseases such as PSC.^44,51,101,102^ This is also the case for MAIT cells within the cholangiocarcinoma microenvironment.^8^ However, MAIT cells appear to be retained specifically within the biliary niche in patients with PSC.^60^ Interestingly, bile from patients with PSC was recently shown to contain MAIT cell antigens.^103^ This, together with evidence showing that cholangiocytes can directly present to and subsequently activate MAIT cells via MR1 and that MAIT cells localise to portal tracts,^8,51^ makes these cells of interest in PSC pathogenesis. Except for cellular cytotoxicity, MAIT cells also possess the capacity to exhibit Th1 and Th17 responses, with the production of IFNγ and IL-17.^104^ However, it remains to be determined whether these cells, in fact, can contribute to early initiation events in PSC or if they instead take part in the propagation of inflammation. Depletion of NK cells in the *Mdr2*^-/-^ mouse model demonstrated the contribution of NK cell-derived IFNγ to sclerosing cholangitis.^105^ However, since total NK cells were depleted in this model, the possible contribution of tissue-resident compared to circulating and/or recruited NK cells remains to be determined. Beyond this, NK cells have also been implicated in the pathogenesis of biliary atresia. They localise close to intrahepatic bile ducts of infants with biliary atresia and can target cholangiocytes via the activating receptor NKG2D in the rotavirus model.^69^ In mice, NK cell activation likely occurs via inflammasome activation, leading to elevated IL-18 levels.^106^ IL-18 has also been identified as a susceptibility gene for biliary atresia,^107^ and the response might be allowed to proceed because of insufficient immune control from regulatory T cells.^108^ However, most of this work on NK cells, both in PSC and biliary atresia, was performed in mouse models. Human translation is warranted, including specific studies of tissue-resident and circulating NK cells. Likewise, we currently have a limited understanding of non-NK cell ILCs and other unconventional T cells in biliary diseases.

## Conclusions and outlook

From studies reviewed above, it is evident that a distinct immunological niche exists in proximity to bile ducts. The important contribution of the immune system to biliary disorders is also clear. The aforementioned insights have raised many new questions. Although novel single-cell approaches reveal cellular heterogeneity at an unprecedented level, consolidation will be desirable with respect to uniform nomenclature as well as the definition of minimally relevant functional populations and/or subsets of immune cells. Clearly, more insights into the contribution of tissue resident compared to recently recruited immune cells (monocytes/macrophages, T cells, innate lymphocytes) in initiation and propagation of biliary disorders are needed. We are only beginning to understand the interplay between immune cells and structural cells (e.g. fibroblasts, cholangiocytes, endothelial cells). Novel technologies will likely greatly aid in this work. Beyond spatial biology, temporal aspects (pre-symptomatic, early, late disease stages) will be of equal importance to consider, especially in human translational studies. In summary, although we have significantly extended our knowledge of biliary immunobiology over the past decade, much remains to be learned (see Box 1 on unresolved issues and proposed research agenda moving forward).

## Box. 1 Unresolved issues and proposed research agenda moving forward

- A detailed picture of the normal distribution and spatial localisation of immune cells along the entire biliary tract system in the context of health would benefit the field as well as serve as an important benchmark for pathogenesis studies.
- Species differences exist between mice and humans with respect to liver and bile duct immune cell populations, the significance of this should be considered in disease pathogenesis studies. Here, cross-species integration of omics data can be of use.
- The biliary niche is distinct compared to surrounding liver parenchyma, however, the exact interplay between cell types within the niche, in health and disease, should be elucidated using newly available technologies.
- Temporal considerations are similarly important, although more challenging in the human setting, to study (early *vs*. late disease) where the former will be of more interest from a disease-specific pathogenesis perspective while the latter might yield insights into mechanisms conserved across disorders.
- Implementation into next-generation precision medicine (diagnostics, prognostics, endpoints in trials, individualised treatment decisions) is the goal and will likely involve considerations and data only attainable from the local spatial biliary niche.

## Abbreviations

CCL: C-C motif chemokine ligand
CCR: C-C motif chemokine receptor
CXCL: C-X-C motif chemokine ligand
CXCR: C-X-C motif chemokine receptor
ERCP: endoscopic retrograde cholangiopancreatography
IFN: interferon
IL-: interleukin
1LC: innate lymphoid cell
MAIT: mucosal-associated invariant T
M1CA/M1CB: MHC-1 chain-related A and B proteins
MR1: MHC class 1 related-1 molecule
NK: natural killer
PBC: primary biliary cholangitis
PSC: primary sclerosing cholangitis
scRNAseq: single-cell RNA sequencing
TGFβ: transforming growth factor-β
Th: T helper
VCAM-1: vascular cell adhesion molecule 1.

## References

[1] Banales JM, Huebert RC, Karlsen T, Strazzabosco M, Larusso NF, Gores GJ (2019). Cholangiocyte pathobiology. Nature.

[2] Sampaziotis F, Muraro D, Tysoe OC, Sawiak S, Beach TE, Godfrey EM (2021). Cholangiocyte organoids can repair bile ducts after transplantation in the human liver. Science.

[3] Karlsen TH, Folseraas T, Thorburn D, Vesterhus M (2017). Primary sclerosing cholangitis - a comprehensive review. J Hepatol.

[4] Hartley JL, Davenport M, Kelly DA (2009). Biliary atresia. Lancet Lond Engl.

[5] Trivedi PJ, Hirschfield GM (2021). Recent advances in clinical practice: epidemiology of autoimmune liver diseases. Gut.

[6] Banales JM, Marin JJG, Lamarca A, Rodrigues PM, Khan SA, Roberts LR (2020). Cholangiocarcinoma 2020: the next horizon in mechanisms and management. Nat Rev Gastroenterol Hepatol.

[7] Ding G-Y, Ma J-Q, Yun J-P, Chen X, Ling Y, Zhang S (2022). Distribution and density of tertiary lymphoid structures predict clinical outcome in intrahepatic cholangiocarcinoma. J Hepatol.

[8] Zimmer CL, Filipovic I, Cornillet M, O’Rourke CJ, Berglin L, Jansson H (2021). Mucosal-associated invariant T-cell tumor infiltration predicts long-term survival in cholangiocarcinoma. Hepatology.

[9] Dong L, Lu D, Chen R, Lin Y, Zhu H, Zhang Z (2021). Proteogenomic characterization identifies clinically relevant subgroups of intrahepatic cholangiocarcinoma. Cancer Cell.

[10] Filipovic I, Sönnerborg I, Strunz B, Friberg D, Cornillet M, Hertwig L (2019). 29-color flow cytometry: unraveling human liver NK cell repertoire diversity. Front Immunol.

[11] Bendall SC, Simonds EF, Qiu P, Amir ED, Krutzik PO, Finck R (2011). Single-cell mass cytometry of differential immune and drug responses across a human hematopoietic continuum. Science.

[12] Picelli S, Björklund ÅK, Faridani OR, Sagasser S, Winberg G, Sandberg R (2013). Smart-seq2 for sensitive full-length transcriptome profiling in single cells. Nat Methods.

[13] Macosko EZ, Basu A, Satija R, Nemesh J, Shekhar K, Goldman M (2015). Highly parallel genome-wide expression profiling of individual cells using nanoliter droplets. Cell.

[14] Aizarani N, Saviano A, Sagar, Mailly L, Durand S, Herman JS (2019). A human liver cell atlas reveals heterogeneity and epithelial progenitors. Nature.

[15] Halpern KB, Shenhav R, Matcovitch-Natan O, Tóth B, Lemze D, Golan M (2017). Single-cell spatial reconstruction reveals global division of labour in the mammalian liver. Nature.

[16] Poch T, Krause J, Casar C, Liwinski T, Glau L, Kaufmann M (2021). Single-cell atlas of hepatic T cells reveals expansion of liver-resident naive-like CD4+ T cells in primary sclerosing cholangitis. J Hepatol.

[17] Wang J, Xu Y, Chen Z, Liang J, Lin Z, Liang H (2020). Liver immune profiling reveals pathogenesis and therapeutics for biliary atresia. Cell.

[18] Zhang M, Yang H, Wan L, Wang Z, Wang H, Ge C (2020). Single cell transcriptomic architecture and intercellular crosstalk of human intrahepatic cholangiocarcinoma. J Hepatol.

[19] Ståhl PL, Salmén F, Vickovic S, Lundmark A, Navarro JF, Magnusson J (2016). Visualization and analysis of gene expression in tissue sections by spatial transcriptomics. Science.

[20] Moses L, Pachter L Museum of spatial transcriptomics. Nat Methods.

[21] Guilliams M, Bonnardel J, Haest B, Vanderborght B, Wagner C, Remmerie A (2022). Spatial proteogenomics reveals distinct and evolutionarily conserved hepatic macrophage niches. Cell.

[22] Hildebrandt F, Andersson A, Saarenpää S, Larsson L, Hul NV, Kanatani S (2021). Spatial Transcriptomics to define transcriptional patterns of zonation and structural components in the mouse liver. Nat Commun.

[23] Goltsev Y, Samusik N, Kennedy-Darling J, Bhate S, Hale M, Vazquez G (2018). Deep profiling of mouse splenic architecture with CODEX multiplexed imaging. Cell.

[24] Ramachandran P, Matchett KP, Dobie R, Wilson-Kanamori JR, Henderson NC (2020). Single-cell technologies in hepatology: new insights into liver biology and disease pathogenesis. Nat Rev Gastroenterol Hepatol.

[25] Schenkel JM, Masopust D (2014). Tissue-resident memory T cells. Immunity.

[26] Björkström NK, Ljunggren H-G, Michaёlsson J (2016). Emerging insights into natural killer cells in human peripheral tissues. Nat Rev Immunol.

[27] Steinert EM, Schenkel JM, Fraser KA, Beura LK, Manlove LS, Igyártó BZ (2015). Quantifying memory CD8 T cells reveals regionalization of immunosurveillance. Cell.

[28] Gasteiger G, Fan X, Dikiy S, Lee SY, Rudensky AY (2015). Tissue residency of innate lymphoid cells in lymphoid and nonlymphoid organs. Science.

[29] Bister J, Guterstam YC, Strunz B, Dumitrescu B, Bhattarai KH, Özenci V (2020). Human endometrial MAIT cells are transiently tissue resident and respond to Neisseria gonorrhoeae. Mucosal Immunol.

[30] Strunz B, Bister J, Jönsson H, Filipovic I, Guterstam YC, Kvedaraite E (2021). Continuous human uterine NK cell differentiation in response to endometrial regeneration and pregnancy. Sci Immunol.

[31] Cuff AO, Robertson FP, Stegmann KA, Pallett LJ, Maini MK, Davidson BR (2016). Eomeshi NK cells in human liver are long-lived and do not recirculate but can be replenished from the circulation. J Immunol.

[32] Pallett LJ, Burton AR, Amin OE, Rodríguez-Tajes S, Patel AA, Zakeri N (2020). Longevity and replenishment of human liver-resident memory T cells and mononuclear phagocytes. J Exp Med.

[33] Zuber J, Shonts B, Lau S-P, Obradovic A, Fu J, Yang S (2016). Bidirectional intragraft alloreactivity drives the repopulation of human intestinal allografts and correlates with clinical outcome. Sci Immunol.

[34] Snyder ME, Finlayson MO, Connors TJ, Dogra P, Senda T, Bush E (2019). Generation and persistence of human tissue-resident memory T cells in lung transplantation. Sci Immunol.

[35] Rosato PC, Beura LK, Masopust D (2016). Tissue resident memory T cells and viral immunity. Curr Opin Virol.

[36] Okła K, Farber DL, Zou W (2021). Tissue-resident memory T cells in tumor immunity and immunotherapy. J Exp Med.

[37] Björkström NK, Kekäläinen E, Mjösberg J (2013). Tissue-specific effector functions of innate lymphoid cells. Immunology.

[38] Blériot C, Chakarov S, Ginhoux F (2020). Determinants of resident tissue macrophage identity and function. Immunity.

[39] Zwicker C, Bujko A, Scott CL (2021). Hepatic macrophage responses in inflammation, a function of plasticity, heterogeneity or both?. Front Immunol.

[40] Racanelli V, Rehermann B (2006). The liver as an immunological organ. Hepatology.

[41] Forkel M, Berglin L, Kekäläinen E, Carlsson A, Svedin E, Michaёlsson J (2017). Composition and functionality of the intrahepatic innate lymphoid cell-compartment in human nonfibrotic and fibrotic livers. Eur J Immunol.

[42] Bruneau A, Guillot A, Tacke F (2022). Macrophages in cholangiopathies. Curr Opin Gastroen.

[43] Provine NM, Klenerman P (2019). MAIT cells in health and disease. Annu Rev Immunol.

[44] Hunter S, Willcox CR, Davey MS, Kasatskaya SA, Jeffery HC, Chudakov DM (2018). Human liver infiltrating γδ T cells are composed of clonally expanded circulating and tissue-resident populations. J Hepatol.

[45] Marquardt N, Béziat V, Nyström S, Hengst J, Ivarsson MA, Kekäläinen E (2015). Cutting edge: identification and characterization of human intra-hepatic CD49a+ NK cells. J Immunol.

[46] Stegmann KA, Robertson F, Hansi N, Gill U, Pallant C, Christophides T (2016). CXCR6 marks a novel subset of T-bet(lo)Eomes(hi) natural killer cells residing in human liver. Sci Rep.

[47] Peng H, Jiang X, Chen Y, Sojka DK, Wei H, Gao X (2013). Liver-resident NK cells confer adaptive immunity in skin-contact inflammation. J Clin Invest.

[48] Burt BM, Plitas G, Zhao Z, Bamboat ZM, Nguyen HM, Dupont B (2009). The lytic potential of human liver NK cells is restricted by their limited expression of inhibitory killer Ig-like receptors. J Immunol.

[49] Dusseaux M, Martin E, Serriari N, Péguillet I, Premel V, Louis D (2011). Human MAIT cells are xenobiotic-resistant, tissue-targeted, CD161hi IL-17-secreting T cells. Blood.

[50] Bandyopadhyay K, Marrero I, Kumar V (2016). NKT cell subsets as key participants in liver physiology and pathology. Cell Mol Immunol.

[51] Jeffery HC, van Wilgenburg B, Kurioka A, Parekh K, Stirling K, Roberts S (2016). Biliary epithelium and liver B cells exposed to bacteria activate intrahepatic MAIT cells through MR1. J Hepatol.

[52] Pallett LJ, Davies J, Colbeck EJ, Robertson F, Hansi N, Easom NJW (2017). IL-2(high) tissue-resident T cells in the human liver: sentinels for hepatotropic infection. J Exp Med.

[53] Wiggins BG, Pallett LJ, Li X, Davies SP, Amin OE, Gill US (2021). The human liver microenvironment shapes the homing and function of CD4+ T-cell populations. Gut.

[54] MacParland SA, Liu JC, Ma X-Z, Innes BT, Bartczak AM, Gage BK (2018). Single cell RNA sequencing of human liver reveals distinct intrahepatic macrophage populations. Nat Commun.

[55] Sierro F, Evrard M, Rizzetto S, Melino M, Mitchell AJ, Florido M (2017). A liver capsular network of monocyte-derived macrophages restricts hepatic dissemination of intraperitoneal bacteria by neutrophil recruitment. Immunity.

[56] Zimmer CL, von Seth E, Buggert M, Strauss O, Hertwig L, Nguyen S (2021). A biliary immune landscape map of primary sclerosing cholangitis reveals a dominant network of neutrophils and tissue-resident T cells. Sci Transl Med.

[57] Thome JJC, Yudanin N, Ohmura Y, Kubota M, Grinshpun B, Sathaliyawala T (2014). Spatial map of human T cell compartmentalization and maintenance over decades of life. Cell.

[58] Milani S, Herbst H, Schuppan D, Stein H, Surrenti C (1991). Transforming growth factors beta 1 and beta 2 are differentially expressed in fibrotic liver disease. Am J Pathol.

[59] Mackay LK, Rahimpour A, Ma JZ, Collins N, Stock AT, Hafon M-L (2013). The developmental pathway for CD103(+)CD8+ tissue-resident memory T cells of skin. Nat Immunol.

[60] von Seth E, Zimmer CL, Reuterwall-Hansson M, Barakat A, Arnelo U, Bergquist A (2018). Primary sclerosing cholangitis leads to dysfunction and loss of MAIT cells. Eur J Immunol.

[61] Fabris L, Perugorria MJ, Mertens J, Björkström NK, Cramer T, Lleo A (2019). The tumour microenvironment and immune milieu of cholangiocarcinoma. Liver Int.

[62] Kruglov EA, Nathanson RA, Nguyen T, Dranoff JA (2006). Secretion of MCP-1/CCL2 by bile duct epithelia induces myofibroblastic transdifferentiation of portal fibroblasts. Am J Physiol-Gastr L.

[63] Govaere O, Cockell S, Haele MV, Wouters J, Delm WV, den Eynde KV (2019). High-throughput sequencing identifies aetiology-dependent differences in ductular reaction in human chronic liver disease. J Pathol.

[64] Heydtmann M, Lalor PF, Eksteen JA, Hübscher SG, Briskin M, Adams DH (2005). CXC chemokine ligand 16 promotes integrin-mediated adhesion of liverinfiltrating lymphocytes to cholangiocytes and hepatocytes within the inflamed human liver. J Immunol.

[65] Isse K, Harada K, Zen Y, Kamihira T, Shimoda S, Harada M (2005). Fractalkine and CX3CR1 are involved in the recruitment of intraepithelial lymphocytes of intrahepatic bile ducts. Hepatology.

[66] Afford SC, Humphreys EH, Reid DT, Russell CL, Banz VM, Oo Y (2014). Vascular cell adhesion molecule 1 expression by biliary epithelium promotes persistence of inflammation by inhibiting effector T-cell apoptosis. Hepatology.

[67] Schrumpf E, Tan C, Karlsen TH, Sponheim J, Björkström NK, Sundnes O (2015). The biliary epithelium presents antigens to and activates natural killer T cells. Hepatology.

[68] Zhang S, Hüe S, Sène D, Penfornis A, Bresson-Hadni S, Kantelip B (2008). Expression of major histocompatibility complex class I chain-related molecule A, NKG2D, and transforming growth factor-b in the liver of humans with alveolar echinococcosis: new actors in the tolerance to parasites?. J Infect Dis.

[69] Shivakumar P, Sabla GE, Whitington P, Chougnet CA, Bezerra JA (2009). Neonatal NK cells target the mouse duct epithelium via Nkg2d and drive tissue-specific injury in experimental biliary atresia. J Clin Invest.

[70] Kvedaraite E (2021). Neutrophil-T cell crosstalk in inflammatory bowel disease. Immunology.

[71] Zweers SJ, Shiryaev A, Komuta M, Vesterhus M, Hov JR, Perugorria MJ (2016). Elevated interleukin-8 in bile of patients with primary sclerosing cholangitis. Liver Int.

[72] Vesterhus M, Holm A, Hov JR, Nygård S, Schrumpf E, Melum E (2017). Novel serum and bile protein markers predict primary sclerosing cholangitis disease severity and prognosis. J Hepatol.

[73] O’Leary CE, Sbierski-Kind J, Kotas ME, Wagner JC, Liang H-E, Schroeder AW (2022). Bile acid-sensitive tuft cells regulate biliary neutrophil influx. Sci Immunol.

[74] Takeuchi M, Vidigal PT, Guerra MT, Hundt MA, Robert ME, Olave-Martinez M (2021). Neutrophils interact with cholangiocytes to cause cholestatic changes in alcoholic hepatitis. Gut.

[75] Kunzmann LK, Schoknecht T, Poch T, Henze L, Stein S, Kriz M (2020). Monocytes as potential mediators of pathogen-induced T-helper 17 differentiation in patients with primary sclerosing cholangitis (PSC). Hepatology.

[76] Guillot A, Guerri L, Feng D, Kim S-J, Ahmed YA, Paloczi J (2021). Bile acid-activated macrophages promote biliary epithelial cell proliferation through integrin αvß6 upregulation following liver injury. J Clin Invest.

[77] Chen Y-Y, Arndtz K, Webb G, Corrigan M, Akiror S, Liaskou E (2019). Intrahepatic macrophage populations in the pathophysiology of primary sclerosing cholangitis. Jhep Rep.

[78] Guicciardi ME, Trussoni CE, Krishnan A, Bronk SF, Pisarello MJL, O’Hara SP (2018). Macrophages contribute to the pathogenesis of sclerosing cholangitis in mice. J Hepatol.

[79] Taylor SA, Chen S-Y, Gadhvi G, Feng L, Gromer KD, Abdala-Valencia H (2021). Transcriptional profiling of pediatric cholestatic livers identifies three distinct macrophage populations. Plos One.

[80] Fabris L, Fiorotto R, Spirli C, Cadamuro M, Mariotti V, Perugorria MJ (2019). Pathobiology of inherited biliary diseases: a roadmap to understand acquired liver diseases. Nat Rev Gastroentero.

[81] Ronca V, Mancuso C, Milani C, Carbone M, Oo YH, Invernizzi P (2020). Immune system and cholangiocytes: a puzzling affair in primary biliary cholangitis. J Leukocyte Biol.

[82] Katt J, Schwinge D, Schoknecht T, Quaas A, Sobottka I, Burandt E (2013). Increased T helper type 17 response to pathogen stimulation in patients with primary sclerosing cholangitis. Hepatology.

[83] Stein S, Henze L, Poch T, Carambia A, Krech T, Preti M (2021). 1L-17A/F enable cholangiocytes to restrict T cell-driven experimental cholangitis by upregulating PD-L1 expression. J Hepatol.

[84] Jeffery HC, Hunter S, Humphreys EH, Bhogal R, Wawman RE, Birtwistle J (2019). Bidirectional cross-talk between biliary epithelium and Th17 cells promotes local Th17 expansion and bile duct proliferation in biliary liver diseases. J Immunol.

[85] Targan SR, Feagan B, Vermeire S, Panaccione R, Melmed GY, Landers C (2016). A randomized, double-blind, placebo-controlled phase 2 study of brodalumab in patients with moderate-to-severe Crohn’s disease. Am J Gastroenterol.

[86] Paillet J, Plantureux C, Lévesque S, Naour JL, Stoll G, Sauvat A (2021). Autoimmunity affecting the biliary tract fuels the immunosurveillance of cholangiocarcinoma. J Exp Med.

[87] Adams DH, Eksteen B (2006). Aberrant homing of mucosal T cells and extra-intestinal manifestations of inflammatory bowel disease. Nat Rev Immunol.

[88] de Krijger M, Wildenberg ME, de Jonge WJ, Ponsioen CY (2019). Return to sender: lymphocyte trafficking mechanisms as contributors to primary sclerosing cholangitis. J Hepatol.

[89] Eksteen B, Mora JR, Haughton EL, Henderson NC, Lee-Turner L, Villablanca EJ (2009). Gut homing receptors on CD8 T cells are retinoic acid dependent and not maintained by liver dendritic or stellate cells. Gastroenterology.

[90] Graham JJ, Mukherjee S, Yuksel M, Mateos RS, Si T, Huang Z (2021). Aberrant hepatic trafficking of gut-derived T cells is not specific to primary sclerosing cholangitis. Hepatology.

[91] Huang B, Lyu Z, Qian Q, Chen Y, Zhang J, Li B (2022). NUDT1 promotes the accumulation and longevity of CD103+ TRM cells in primary biliary cholangitis. J Hepatol.

[92] Jiang X, Bergquist A, Löscher B-S, Venkatesh G, Mold JE, Holm K (2021). A heterozygous germline CD100 mutation in a family with primary sclerosing cholangitis. Sci Transl Med.

[93] Cargill T, Culver EL (2021). The role of B cells and B cell therapies in immune-mediated liver diseases. Front Immunol.

[94] Macpherson AJ, Hunziker L, McCoy K, Lamarre A (2001). IgA responses in the intestinal mucosa against pathogenic and non-pathogenic microorganisms. Microbes Infect.

[95] Löhr J-M, Vujasinovic M, Rosendahl J, Stone JH, Beuers U (2022). IgG4-related diseases of the digestive tract. Nat Rev Gastroentero.

[96] Hubers LM, Schuurman AR, Buijs J, Mostafavi N, Bruno MJ, Vermeulen RCH (2021). Blue-collar work is a risk factor for developing IgG4-related disease of the biliary tract and pancreas. Jhep Rep.

[97] Bednarek J, Traxinger B, Brigham D, Roach J, Orlicky D, Wang D (2018). Cytokine-producing B cells promote immune-mediated bile duct injury in murine biliary atresia. Hepatology.

[98] Chung BK, Henriksen EKK, Jørgensen KK, Karlsen TH, Hirschfield GM, Liaskou E (2018). Gut and liver B cells of common clonal origin in primary sclerosing cholangitis-inflammatory bowel disease. Hepatol Commun.

[99] Palm NW, de Zoete MR, Cullen TW, Barry NA, Stefanowski J, Hao L (2014). Immunoglobulin A coating identifies colitogenic bacteria in inflammatory bowel disease. Cell.

[100] Xu GJ, Kula T, Xu Q, Li MZ, Vernon SD, Ndung’u T (2015). Viral immunology. Comprehensive serological profiling of human populations using a synthetic human virome. Science.

[101] Riva A, Patel V, Kurioka A, Jeffery HC, Wright G, Tarff S (2017). Mucosa-associated invariant T cells link intestinal immunity with antibacterial immune defects in alcoholic liver disease. Gut.

[102] Niehaus CE, Strunz B, Cornillet M, Falk CS, Schnieders A, Maasoumy B (2020). MAIT cells are enriched and highly functional in ascites of patients with decompensated liver cirrhosis. Hepatology.

[103] Valestrand L, Zheng F, Hansen SH, Øgaard J, Hov JR, Björkström NK (2022). Bile from patients with primary sclerosing cholangitis contains mucosal-associated invariant T cell antigens. Am J Pathol.

[104] Dias J, Boulouis C, Gorin J-B, van den Biggelaar RHGA, Lal KG, Gibbs A (2018). The CD4-CD8- MAIT cell subpopulation is a functionally distinct subset developmentally related to the main CD8+ MAIT cell pool. Proc Natl Acad Sci.

[105] Ravichandran G, Neumann K, Berkhout LK, Weidemann S, Langeneckert AE, Schwinge D (2019). Interferon-γ-dependent immune responses contribute to the pathogenesis of sclerosing cholangitis in mice. J Hepatol.

[106] Yang L, Mizuochi T, Shivakumar P, Mourya R, Luo Z, Gutta S (2018). Regulation of epithelial injury and bile duct obstruction by NLRP3IL-1R1 in experimental biliary atresia. J Hepatol.

[107] Liang J, Wen Z, Zhao J, Liang Q, Liu T, Xia H (2018). Association of IL18 genetic polymorphisms with increased risk of biliary atresia susceptibility in Southern Chinese children. Gene.

[108] Miethke AG, Saxena V, Shivakumar P, Sabla GE, Simmons J, Chougnet CA (2010). Post-natal paucity of regulatory T cells and control of NK cell activation in experimental biliary atresia. J Hepatol.

[109] Vivier E, Artis D, Colonna M, Diefenbach A, Santo JPD, Eberl G (2018). Innate lymphoid cells: 10 years on. Cell.

[110] Godfrey DI, Uldrich AP, McCluskey J, Rossjohn J, Moody DB (2015). The burgeoning family of unconventional T cells. Nat Immunol.

[111] Ramachandran P, Dobie R, Wilson-Kanamori JR, Dora EF, Henderson BEP, Luu NT (2019). Resolving the fibrotic niche of human liver cirrhosis at singlecell level. Nature.

[112] Scott CL, Zheng F, Baetselier PD, Martens L, Saeys Y, Prijck SD (2016). Bone marrow-derived monocytes give rise to self-renewing and fully differentiated Kupffer cells. Nat Commun.

[113] Remmerie A, Martens L, Thoné T, Castoldi A, Seurinck R, Pavie B (2020). Osteopontin expression identifies a subset of recruited macrophages distinct from Kupffer cells in the fatty liver. Immunity.

